# Clinical *Talaromyces marneffei* isolates exhibit enhanced stress tolerance and diminished antifungal responsiveness relative to non-clinical strains

**DOI:** 10.3389/ffunb.2026.1838799

**Published:** 2026-05-05

**Authors:** Kai-Su Pan, Siwen Jiang, Dong-Yan Zheng, Yan-Qing Zheng, Wei-Xuan Wu, Cun-Wei Cao

**Affiliations:** 1 Department of Dermatology and Venereology, The First Affiliated Hospital of Guangxi Medical University, Nanning, Guangxi, China; 2 Guangxi Key Laboratory of Mycosis Research and Prevention, Nanning, China; 3 Fourth People’s Hospital of Nanning, Nanning, China; 4 Fangchenggang Wanqing Institute of Mycosis Prevention and Control, Fangchenggang, China

**Keywords:** antifungal, clinical strains, HIV, non-clinical strains, *Talaromyces marneffei*, talaromycosis

## Abstract

**Introduction:**

Talaromycosis, caused by the dimorphic fungus *Talaromyces marneffei*, is a significant opportunistic infection in HIV/AIDS patients in Southeast Asia. This study aimed to investigate the antifungal susceptibility of clinical *T. marneffei* strains (from HIV-positive and HIV-negative patients) and non-clinical strains, and to compare their adaptability to various stress conditions.

**Methods:**

A total of 196 *T. marneffei* strains were assessed using broth microdilution and Sensititre YeastOne YO10 methods to determine MICs against nine antifungal agents. Stress adaptability was evaluated using 20 randomly selected non-clinical and 20 clinical strains incubated under KCl, Congo red (CR), H_2_O_2_, and Calcofluor white (CFW).

**Results:**

Azole antifungals (itraconazole, voriconazole, posaconazole) exhibited low MICs, indicating strong activity; Clinical strains showed higher geometric mean MICs for most antifungals than non-clinical strains. Non-clinical strains showed significantly less adaptability to stress than clinical strains under CR, H_2_O_2_, and CFW (p<0.05), with smaller colony diameters and slower growth.

**Discussion:**

These findings suggest that non-clinical strains may undergo phenotypic adaptations when infecting human hosts, enhancing their resilience to environmental factors and immune pressures. Antifungal susceptibility was not associated with HIV status or patient age, but linked to host adaptation.

## Introduction

Talaromycosis is a fungal infection that is highly prevalent in northern Thailand, Vietnam, Myanmar, north-eastern India, and southern China, caused by the dimorphic fungus *Talaromyces marneffei* (*T. marneffei*). It is a notable opportunistic infection of HIV/AIDS patients in endemic regions (Vanittanakom et al., 2006; Huang et al., 2015; Chan et al., 2016; Chen et al., 2017; He et al., 2021). Despite decades of research on the epidemiology and ecology of *T. marneffei*, the fundamental ecology of this unique pathogen remains poorly understood. Bamboo rats are recognized as animal hosts for *T. marneffei*, and currently, isolating *T. marneffei* from the environment poses significant challenges (Gugnani et al., 2004). Our previous study (Cao et al., 2011) demonstrated that *T. marneffei* strains infecting humans and rats share the same genotype, suggesting a common environmental source of infection. Moreover, the findings indicated that small mammals play an essential role in the life cycle of *T. marneffei*, phenotypic divergence between clinical and environmental isolates is well documented in dimorphic fungal pathogens (Boyce and Andrianopoulos, 2015; Pan et al., 2022). However, it remains unclear whether *T. marneffei* undergoes phenotypic adaptation changes following infections in humans or bamboo rats when subjected to different selective pressures, such as adaptation to various hosts, which may influence the strains’ responses to antifungal treatments and environmental stressors. This study aims to evaluate clinical and non-clinical strains through *in vitro* antifungal susceptibility testing while also assessing their growth under various environmental conditions. Additionally, we compared the broth microdilution and Sensititre YeastOne YO10 methods for antifungal susceptibility testing.

## Materials and methods

### Strains

A total of 176 isolates were derived from clinical patients, with 120 sourced from HIV-negative individuals (including 12 children) and 56 from HIV-positive individuals. Additionally, 20 non-clinical strains of *T. marneffei* were collected, including 16 strains obtained from bamboo rats, which included the wild-type strain FRR 2161 (which was generously provided by Professor Alex Andrianopoulos, Department of Genetics, University of Melbourne, Australia), and 4 strains isolated from soil samples collected around bamboo rat caves (see **Table 1**). All isolates were stored and analyzed at the Department of Dermatology and Venereology, First Affiliated Hospital of Guangxi Medical University. This study was approved by the Medical Ethics Committee of the First Affiliated Hospital of Guangxi Medical University (approval number: 2023-K076-01). The quality control strains used were *Candida parapsilosis* ATCC 22019 and *Candida krusei* ATCC 6258. The antifungal sensitivity of these isolates was assessed using the yeast phase of *T. marneffei*, the pathogenic form of the fungus that grows optimally at 37 °C. Yeast cells were prepared following our previously reported method (Cao et al., 2009). In brief, the isolates were cultured on brain heart infusion agar (BHI) at 37 °C for 3 to 7 days until adequate growth was achieved. The resulting yeast-like cells were then harvested, resuspended in sterile water, and vortexed thoroughly to ensure homogeneity. The concentration of yeast cells was quantified using a hemocytometer in conjunction with lactophenol-cotton blue staining. Subsequently, the yeast cell suspensions were added to RPMI 1640 (Roswell Park Memorial Institute 1640) medium to prepare a stock solution with a concentration of 1-5 × 10^6^ CFU/ml. This stock solution was then diluted 100-fold to create a working solution with a final concentration of 1-5 × 10^4^ CFU/ml for antifungal susceptibility testing.

**Table 1:** Summary of information regarding *Talaromyces marneffei* used in this study.

Types of strains	Sources and information	Total
non-clinical strains	bamboo rat	16
	Soil around the bamboo rat cave	4
clinical strains	people with HIV(All were older than 18 years)	56
	people without HIV (older than 18 years)	108
	people without HIV (under 18 years old)	12
		196

### Antifungal agents and other reagents

The antifungal agents utilized in this study included fluconazole (FLC), itraconazole (ITC), voriconazole (VOC), posaconazole (POS), isavuconazole (ISA), amphotericin B (AMB), caspofungin (CAS), micafungin (MCA), and anidulafungin (ANI). All compounds were sourced as pure powders from Sigma-Aldrich (USA). For preparation, water-insoluble agents including AMB, ITC, POS, VOC, and ANI were dissolved in 100% dimethyl sulfoxide (DMSO). Conversely, water-soluble compounds including FLC, ISA, CAS, and MCA were prepared in sterile distilled water. The stock solutions of these antifungal agents were then diluted in RPMI 1640 medium (Gibco, USA) and subsequently subjected to two-fold serial dilutions to obtain the final concentrations required for the antifungal susceptibility assays.

### Antifungal susceptibility assays

Antifungal susceptibility profiles were evaluated using the broth microdilution method in accordance with Clinical and Laboratory Standards Institute (CLSI) guidelines, specifically documents M27-A4, and previously published studies (Cao et al., 2009; Lau et al., 2017; Wayne, 2017; Wayne, 2020). The final drug concentrations tested were as follows: 0.125 to 64 μg/ml for FLC, CAS, MCA, and ANI; 0.002 to 1 μg/ml for ITC, VOC, POS, and ISA; and 0.03 to 16 μg/ml for AMB (Cao et al., 2009; Luo et al., 2019). These concentrations were selected based on the susceptibility profiles of *T. marneffei* to these antifungal agents. The plates were incubated at 37 °C for 72 hours. The minimum inhibitory concentrations (MICs) for AMB were defined as the lowest drug concentration that showed no visible growth, whereas for the other drugs, the MICs were determined as the lowest concentrations that inhibited 50% of growth compared to the control. Each assay was performed in duplicate on separate days to ensure reproducibility.

Additionally, the Sensititre YeastOne YO10 assay was employed to assess fungal sensitivity following the manufacturer’s instructions. Disposable Sensititre panels, consisting of 96-well plates containing alamarBlue for colorimetric determination, were used for antifungal susceptibility analysis of the 196 strains. After 72 hours of incubation at 37°C, the MIC was determined by measuring the color change of the alamarBlue reagent. A red color indicates fungal growth, while a blue color indicates no growth. The MIC values were visually interpreted and recorded as the lowest drug concentration causing 50% inhibition or complete inhibition (for AMB) of fungal growth.

### Stress exploration experiment

To investigate the sensitivity of non-clinical strains and clinical strains of *T. marneffei* to salt stress, oxidative stress, and high osmolarity conditions, 20 non-clinical strains and 20 clinical strains were randomly selected from the total 196 isolates. Random sampling was employed to minimize selection bias and ensure representative analysis. These strains were cultured on ANM containing different concentrations of potassium chloride (KCl), hydrogen peroxide (H_2_O_2_), Congo red (CR), or Calcofluor white (CFW). The inoculation concentration was standardized to 10^5^ CFU/ml conidia. Stress assays were performed as previously described for *T. marneffei (*Pan et al., 2022; Du et al., 2024). The plates were incubated at 25 °C for 14 days. During this period, the colony diameters were measured daily to monitor growth response under the different stress conditions. All operations were carried out in the biological safety cabinet. Statistical analysis of colony diameters was performed using independent sample Mann-Whitney U tests, as clinical and non-clinical strains represented independent groups. Bonferroni correction was applied to account for multiple comparisons across different stress conditions.

## Results

### Antifungal susceptibility profiles of *T. marneffei* isolates

The MICs, MIC_50_, MIC_90_, and geometric mean (GM) distributions of the nine antifungal agents against 196 strains of *T. marneffei*, derived from both non-clinical and patient sources, are presented in **Table 2**. 95% confidence intervals (95% CIs) were calculated for GM MIC comparisons between groups. Analysis of the strains indicated that the GM MIC of non-clinical strains was generally lower than that for clinical strains, with the exceptions of POS and ISA. Notably, many clinical strains exhibited higher MICs compared to non-clinical strains.

**Table 2:** *In vitro* MICs determinations of nine antifungal agents against non-clinical and clinical strains.

Antifungal	Types of strains	MIC Range (μg/ml)	MIC_50_ (μg/ml)	MIC_90_ (μg/ml)	GM (μg/ml)	95% CI
FLC	non-clinical Strains	0.25-4	1	2	1.24	1.12–1.36
	clinical Strains	0.25-16	2	4	2.72	2.45–3.00
ITC	non-clinical Strains	0.008-0.015	0.008	0.015	0.0095	0.0087–0.0105
	clinical Strains	0.008-0.06	0.015	0.03	0.016	0.0144–0.0176
VOC	non-clinical Strains	0.004-0.015	0.004	0.003	0.0038	0.0034–0.0042
	clinical Strains	0.002-0.03	0.004	0.015	0.0075	0.0068–0.0083
POS	non-clinical Strains	0.002-0.008	0.002	0.004	0.0026	0.0023–0.0028
	clinical Strains	0.002-0.008	0.002	0.004	0.0037	0.0031–0.0041
ISA	non-clinical Strains	0.002-0.03	0.002	0.002	0.0021	0.0018–0.0023
	clinical Strains	0.002-0.015	0.002	0.004	0.0023	0.0021–0.0025
AMB	non-clinical Strains	1-2	1	2	1.01	0.93–1.11
	clinical Strains	0.25-4	1	2	1.37	1.23–1.51
CAS	non-clinical Strains	8-16	8	16	10.56	9.54–11.62
	clinical Strains	8-32	16	32	14.84	13.36–16.32
MCA	non-clinical Strains	8-16	8	16	10.89	9.83–11.98
	clinical Strains	8-32	16	32	13.43	12.09–14.76
ANI	non-clinical Strains	4-16	4	8	6.50	5.85–7.18
	clinical Strains	4-32	8	16	11.58	10.42–12.77

FLC, Fluconazole; ITC, Itraconazole; VOC, Voriconazole; POS, Posaconazole; ISA, Isavuconazole; AMB, Amphotericin B; CAS, Caspofungin; MCA, Micafungin; ANI, Anidulafungin; GM, Geometric Mean; MIC50, Minimum inhibitory concentration required to inhibit the growth of 50% of organisms; MIC90, Minimum inhibitory concentration required to inhibit the growth of 90% of organisms; 95% CI, 95% confidence interval.

### Stress response phenotypes between non-clinical and clinical *T. marneffei* strains

**Figure 1:**
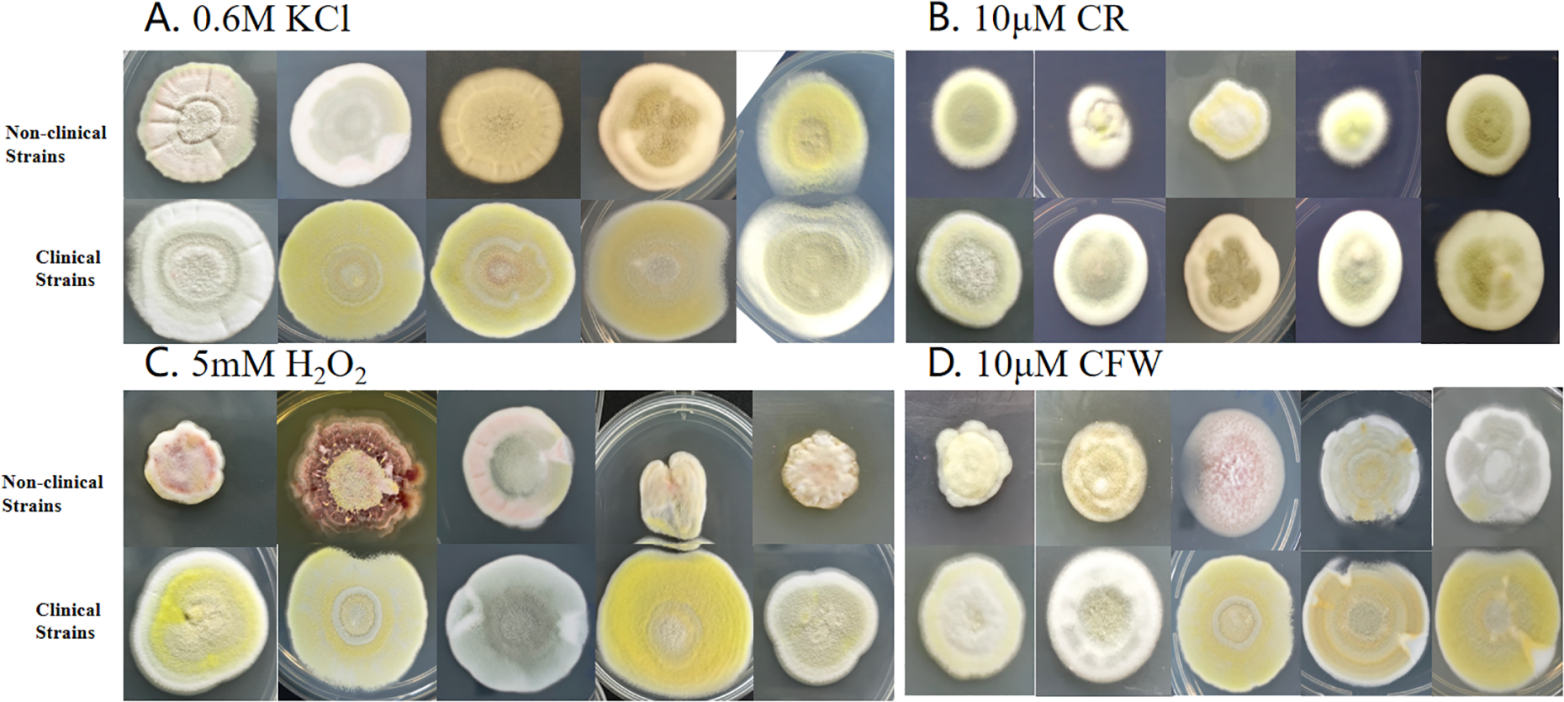
Growth of non-clinical and patients strains under different pressure stresses. **(A)** 0.6M KCI, **(B)** 10μM CR, **(C)** 5mM H_2_O_2_ and **(D)** 10μM CFW ANM medium at 25°C for 14 days. The results indicate that non-clinical strains grew slowly than patients strains.

**Figure 2:**
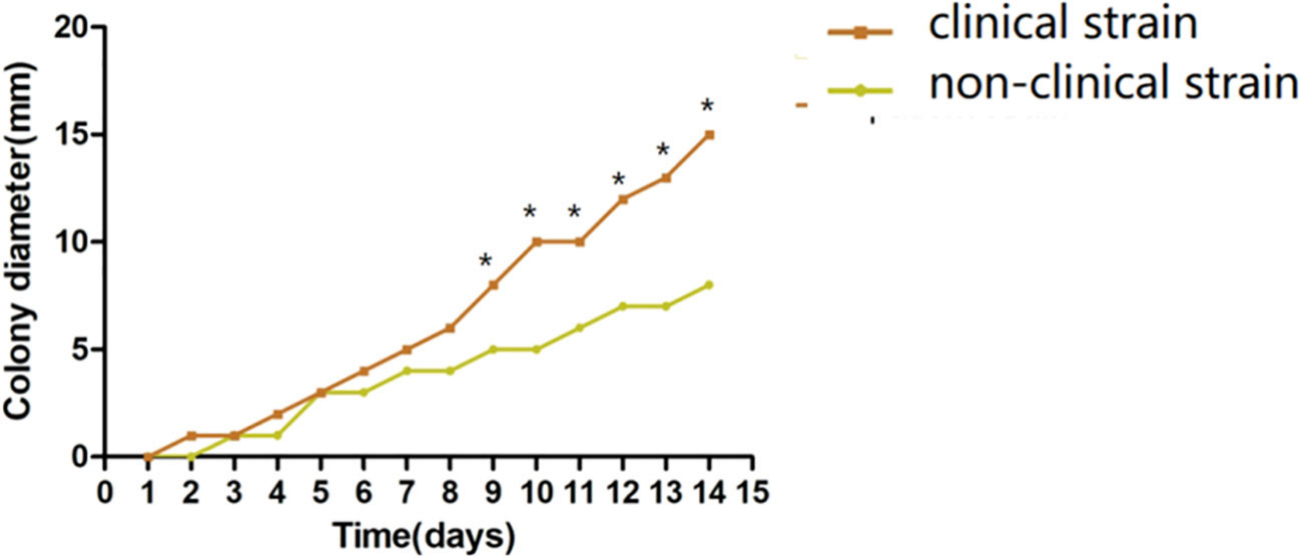
Growth comparison between non-clinical and clinical *T. marneffei* strains under stress conditions. Conidia from 20 non-clinical and 20 clinical strains were inoculated on ANM medium and incubated at 25 °C for 14 days. Colony diameters were measured daily. Data are presented as the mean ± standard deviation (SD). Statistical analysis was performed using the Mann-Whitney U test with Bonferroni correction for multiple comparisons. The asterisk (*) denotes a statistically significant difference (*p* < 0.05) in colony diameter between non-clinical and clinical Talaromyces marneffei strains under stress conditions, as determined by the Mann–Whitney U test with Bonferroni correction.

Results from the stress experiments revealed that non-clinical strains were generally less adaptable to various stress conditions compared to clinical strains. As shown in **Figure 1**, on ANM solid medium supplemented with KCl, CR, H_2_O_2_, and CFW, non-clinical strains exhibited smaller colony diameters. Notably, under H_2_O_2_ stress, these strains displayed irregular colony morphologies and stress-induced pigment production, suggesting distinct stress response mechanisms that warrant further investigation. **Figure 2** presents the average colony diameters of 10 non-clinical and 10 clinical strains at different time points. After 14 days of incubation, non-clinical strains showed significantly slower growth and smaller colonies under conditions containing 10 µM CR, 5 mM H_2_O_2_, and 10 µM CFW (*p < 0.05*). Effect sizes (r values) for these comparisons ranged from 0.35 to 0.52, indicating moderate to large phenotypic differences between groups. A similar trend was observed under 0.6 M KCl stress, though the difference did not reach statistical significance.

### Comparison of broth microdilution and sensititre yeastone YO10 methods

The MICs of nine antifungal agents against *T. marneffei* strains, determined using broth microdilution and Sensititre YeastOne YO10 methods, are summarized in **Table 3**. All MICs for control strains fell within the expected range. In the yeast phase of *T. marneffei*, the MICs for azoles were notably low, ranging from 0.008 - 0.03/0.008 - 0.06 µg/ml for ITC, 0.004 - 0.03/0.002 - 0.015 µg/ml for VOC, and 0.002 - 0.008/0.002 - 0.004 µg/ml for POS, except for FLC, which ranged from 0.25 - 16/0.5 - 8 µg/ml. In contrast, the MICs for CAS, MCA, and ANI were significantly higher. For AMB, the MICs varied from 0.5 - 4/0.25 - 2 µg/ml. The essential agreements (EAs) within ± 2 dilutions between the broth microdilution and Sensititre YeastOne YO10 methods were as follows: AMB (97.3%), ITC (95.7%), POS (96.8%), FLC (96.8%), VOC (96.2%), CAS (99.5%), MCA (97.3%), and ANI (91.4%). These findings suggest that the azoles ITC, VOC, and POS demonstrate effective antifungal activity against the 196 strains of *T. marneffei*, while echinocandins exhibited markedly higher MICs against the yeast phase of *T. marneffei*.

**Table 3:** *In vitro* MIC determinations of eight antifungal agents against *T. marneffei* strains as determined by both broth microdilution and Sensititre YeastOne YO10 (μg/ml).

l and test method	FLC	ITC	VOC	POS	AMB	CAS	MCA	ANI
Broth Microdilution	0.25-16	0.008-0.03	0.004-0.03	0.002-0.008	0.5-4	8-32	8-32	4-16
Sensititre YeastOne YO10	0.5-8	0.008-0.06	0.002-0.015	0.002-0.004	0.25-2	8-16	8-16	4-32
EA ± 1(%)	84.9	86	90.7	84.4	89.2	87.9	90.3	80.6
EA ± 2(%)	96.8	95.7	96.2	96.8	97.3	99.5	97.3	91.4

FLC, fluconazole; ITC, itraconazole; VOC, voriconazole; POS, posaconazole; ISA, isavuconazole; AMB, Amphotericin B; CAS, caspofungin; MCA, micafungin; ANI, Anidungin. EA, Essential agreement (EA =MICs within ± 1 dilution/twofold dilution of reference MICs.).

## Discussion

A total of 196 *T. marneffei* strains were analyzed in this study, of which 108 were from HIV-positive patients, 56 were from HIV-negative adults, 12 were from HIV-negative children, and 20 were non-clinical strains. Overall, MIC values of strains from HIV-positive patients, HIV-negative adults, and HIV-negative children were generally comparable, and no significant differences were observed for all nine antifungals tested in these three clinical subgroups. This suggests that antifungal susceptibility of *T. marneffei* is not significant associated with HIV status or patient age (adult vs. Children).

However, there are clear differences between clinical strains, irrespective of HIV or age, and non-clinical strains. The majority of clinical isolates had higher GM MIC values than non-clinical isolates, indicating a trend toward decreased GM susceptibility in strains that have adapted to the human host. Similar phenotypic divergence between clinical and environmental strains has been reported in other pathogenic fungi (Boyce and Andrianopoulos, 2015; Horta et al., 2022).These findings suggest that the observed changes in antifungal susceptibility are more closely related to host adaptation, both clinical and non-clinical sources, than to HIV infection status or patient age.

Currently, the available guideline for the treatment of *T. marneffei* infection only mentions AMB, ITC and VOC (Thompson et al., 2021). Our study provides new insights into the antifungal susceptibility of *T. marneffei*, particularly emphasizing the pathogen’s high sensitivity to ISA, an antifungal agent not yet recommended for the treatment of talaromycosis. This finding suggests a potential clinical application for ISA in the treatment of *T. marneffei*, thereby expanding therapeutic options beyond the currently recommended agents, AMB, ITC and VOC.

Our stress experiments indicated that non-clinical strains of *T. marneffei* demonstrated reduced stress tolerance compared to clinical strains. The slower growth and smaller colony diameters under stress conditions, including CR, H_2_O_2_, and CFW exposure (*p < 0.05*), suggest that clinical isolates may have developed phenotypic adaptations to better withstand the pressures imposed by the human immune system. These characteristics are consistent with host-specific phenotypic adaptation during infection, though the current study did not perform genomic or molecular analyses to elucidate the underlying molecular mechanisms. The enhanced stress tolerance observed in clinical strains is likely driven by selective pressures associated with survival and proliferation within human tissues. Notably, bamboo rats serves as natural hosts of *T. marneffei*. However, it remains unclear whether this adaptation occurred within the host during the infection process or in the environment prior to the strain infecting a human. The comparison of strains isolated from bamboo rats versus humans under adverse conditions has not been previously addressed, and future studies could help clarify whether the observed resistance traits were acquired in the patient or were pre-existing in the environment, thus contributing to the pathogenic potential of *T. marneffei*. While our findings indicate that these strains may have initially been infected from the same environment, they also suggest a potential phenotypic adaptation relationship between the two sources, possibly linked to the phenotypic adaptation of drug tolerance in response to human immune pressure.

Most previous studies have employed the broth microdilution method in accordance with CLSI guidelines (Pan et al., 2022). Although this method is reliable, it can be time-consuming and sometimes problematic due to trailing endpoints. The E-test method, while simpler, often faces challenges related to the uniform growth of the yeast phase of *T. marneffei* (Lau et al., 2017). In contrast, the Sensititre YeastOne YO10 method (Espinel-Ingroff et al., 2004; Lo et al., 2025) provides a straightforward approach with easily interpretable results. Our findings confirmed the high efficacy of the Sensititre YeastOne YO10 method, revealing a high essential agreement (EA ± 2, ranging from 91.4% to 99.5%) with the broth microdilution method. However, it is important to note that the Sensititre method uses a different endpoint, where no growth (indicated by a blue color) is interpreted as complete inhibition, which contrasts with the CLSI broth microdilution method, where a 50% reduction in growth is used as the endpoint (i.e., partial inhibition, not complete inhibition). This stricter endpoint in the Sensititre method may have influenced the high essential agreement observed. Previous studies, such as Lei et al. (Lei et al., 2018) have also validated the efficacy of the Sensititre YeastOne YO10 method for assessing the susceptibility of *T. marneffei* to antifungal agents, though they similarly acknowledged that partial inhibition (where the color transition begins) is the recommended endpoint for yeast when using the Sensititre method. The addition of the colorimetric dye in the Sensititre method is the main distinction between it and the CLSI method, but both methods assess antifungal activity in a similar manner. Therefore, the observed essential agreement should be interpreted with this difference in mind, and further studies using the recommended partial inhibition endpoint for Sensititre may provide a more accurate comparison.

It should be noted that this study has some limitations. First, the phenotypic differences observed between non-clinical and clinical strains have not been validated by genomic or molecular analyses, and the underlying mechanisms need to be elucidated in further studies. Secondly, because the host immune status, clinical medication and other factors will affect the prognosis of patients, the *in vitro* MIC results cannot fully reflect the actual efficacy of *in vivo* treatment. In addition, the single-center design of this study may limit the generalizability of the findings to other geographic regions. In the future, it is necessary to combine genomic analysis to further elucidate the molecular mechanism of adaptive evolution and drug resistance characteristics of clinical strains of *T. marneffei*, so as to provide more comprehensive evidence-based basis for clinical diagnosis and treatment.

## Data Availability

The original contributions presented in the study are included in the article/**Supplementary Material**. Further inquiries can be directed to the corresponding author.
